# Effect of uniform capacitively coupled electric fields on matrix metabolism of osteoarthritic cartilage

**DOI:** 10.1186/s42234-022-00096-w

**Published:** 2022-09-14

**Authors:** Kaleb Noruzi, Pooja Swami, Lidia Frejo, Jason Wright, Jason Wong, Daniel Grande, Timir Datta-Chaudhuri

**Affiliations:** 1 Orthopaedic Research Laboratory, Feinstein Institutes for Medical Research, 350 Community Dr, Manhasset, NY 11030 USA; 2 New York Medical College, 40 Sunshine Cottage Rd, Valhalla, NY 10595 USA; 3 Department of Genomic Medicine, Otology and Neurotology Group CTS495, GENYO, Centre for Genomics and Oncological Research, Pfizer University of Granada Andalusian Regional Government, PTS, 18016 Granada, Spain; 4 Sensorineural Pathology Programme, Centro de Investigación Biomédica en Red en Enfermedades Raras, CIBERER, 28029 Madrid, Spain; 5 Department of Otolaryngology, Instituto de Investigación Biosanitaria Ibs.Granada, Hospital Universitario Virgen de Las Nieves, Universidad de Granada, 18014 Granada, Spain; 6 Institute for Bioelectronic Medicine, Feinstein Institutes for Medical Research, 350 Community Dr, Manhasset, NY 11030 USA

**Keywords:** Osteoarthritis, Articular cartilage, Chondrocytes, Cartilage explants, Capacitively coupled, Electric stimulation

## Abstract

**Background:**

Osteoarthritis (OA) is a common and debilitating condition characterized by degeneration of hyaline cartilage. Currently, there is no treatment for OA that directly targets degradation of cartilage matrix. Capacitively coupled electric fields (CCEFs) represent a noninvasive and cost-effective treatment modality that can potentially restore articular cartilage homeostasis. Previous studies showed that stimulation of articular cartilage with CCEFs resulted in upregulation of anabolic factors and downregulation of catabolic factors. These studies didn’t explain the derivation of the CCEFs or verify their uniformity and field strength, so it’s possible that cartilage wasn’t exposed to uniform field strength. The present study aims to employ CCEFs with verified uniform field strength in two *in-vitro* models of OA to investigate its potential to preserve cartilage matrix and validate the results of the aforementioned studies.

**Methods:**

Rabbit hyaline chondrocytes and full-thickness bovine articular cartilage explants were cultured in the absence or presence of CCEF and in the absence or presence of Interleukin1-B (IL-1B). Quantitative polymerase chain reaction (QPCR) was performed on chondrocytes to measure gene expression of ADAM-TS4, MMP3, MMP9, IL-6, TIMP1, and TIMP2. QPCR was performed on explants to measure gene expression of MMP3, Aggrecan, Collagen-2, and TIMP1. Aggrecan concentration in explants was measured with histology. Statistical analysis was performed using one-way analysis of variance and Tukey–Kramer multiple comparison test.

**Results:**

The treatment of chondrocytes with IL-1B resulted in upregulated expression of ADAM-TS4, MMP3, MMP9, and IL-6, while simultaneous administration of IL-1B and CCEF led to a relative decrease in ADAM-TS4, MMP3, MMP9, and IL-6 expression and a relative increase in TIMP1 and TIMP2 expression. Application of IL-1B and CCEF to the explants resulted in decreased expression of MMP3 and increased expression of Aggrecan, Collagen-2, and TIMP1 when compared to application of IL-1B alone.

**Conclusion:**

The data indicate that application of a CCEF with verified uniformity may result in upregulation of cartilage anabolic factors even in the presence of IL-1B while attenuating IL-1B induced upregulation of catabolic factors in both monolayer culture and whole tissue. These results demonstrate the potential of CCEFs to suppress the progression of OA and regenerate articular cartilage matrix.

## Background

Osteoarthritis (OA) is the most common form of arthritis. It develops when the protective hyaline cartilage on the ends of bones wears down over time. An estimated 27 million adults have OA in the United States (Neogi 2014). Prevalence of OA is higher among women and reportedly increasing globally due to higher life expectancy of populations, obesity, and other factors (Palazzo et al. 2016).

OA causes pain, inflammation, and reduced motion in all joints, particularly in the joints of the knees, hips, shoulders, hands, and vertebral column (Palazzo et al. 2016). These symptoms can be temporarily managed by routine modalities such as pain medication and injection of steroids and hyaluronic acid into joints. Joint replacements are the last alternative as they are invasive and costly (Hunter and Bierma-Zeinstra 2019). None of these treatments directly address the disease process of OA, which is an imbalance between cartilage matrix synthesis and degradation (Hunter and Bierma-Zeinstra 2019; Brighton et al. 2008).

Advanced cell-based therapies have sought to regenerate damaged cartilage and restore the normal functions of joints. The use of autologous mesenchymal stem cells in these therapies have been limited by highly invasive harvesting procedures, limited collection sites, and high cost (Madeira et al. 2014). Autologous chondrocytes were once considered the best candidate for cartilage regenerative therapies, but they have faced several challenges including the invasiveness of cartilage biopsy, a two-step surgical procedure, lack of proliferation vigor, and loss of phenotype (Madeira et al. 2014; Zingler et al. 2016; Ma et al. 2013).

The application of electric fields in regenerative therapies has shown potential as a non-invasive and cost-effective option for the healing of bone, tendon, and cartilage (Brighton et al. 2008; Tucker et al. 2017; Vinhas et al. 2019; Wang et al. 2004; Brighton et al. 2013; Brighton et al. 2006). The FDA has approved use of electrical fields in the treatment of long-bone fracture non-unions and as an adjunct to spinal fusion surgery (Tucker et al. 2017). Capacitively coupled electrical fields (CCEFs) represent a method of supplying a defined electrical signal to tissue or cells. They are created by placing the tissue or cells between two electrodes, forming a capacitor or device that stores a charge (Brighton et al. 2008).

Healthy articular cartilage is maintained by a balance of matrix synthesis and degradation mediated by chondrocytes based on their response to various biological signals. Mechanical deformation of articular cartilage during joint loading produces electrical signals that are believed to influence the anabolic and catabolic activity of chondrocytes and therefore contribute to regulating cartilage homeostasis. Electrical stimulation of osteoarthritic articular cartilage with CCEFs may restore homeostasis and preserve matrix by simulating these natural electrical signals (Brighton et al. 2008; Wang et al. 2004).

Brighton and coauthors have demonstrated that CCEFs promote cartilage matrix anabolism while decreasing cartilage matrix catabolism in both normal and osteoarthritic articular cartilage (Brighton et al. 2008; Wang et al. 2004; Brighton et al. 2013; Brighton et al. 2006). In these experiments, Interleukin1-B (IL-1B), an inflammatory cytokine, was used to simulate a degradative environment mimicking OA as it downregulates synthesis of cartilage matrix components and upregulates matrix metalloproteinases (MMPs) that catabolize matrix (Brighton et al. 2008; Brighton et al. 2013; Brighton et al. 2006; Martel-Pelletier et al. 2011). The strength of the electric field applied to cartilage was stated to be 20 mV/cm (Brighton et al. 2008; Wang et al. 2004; Brighton et al. 2013; Brighton et al. 2006). However, there was not a clear explanation of the derivation of the electrical field generated by the experimental setup in these experiments and field strength level and uniformity were not verified through analysis, so it is possible that the cartilage was not exposed to uniform field strength of 20 mV/cm as stated. Cartilage may respond differently to different CCEF field strengths. We believed that it was necessary to apply CCEFs with field strengths that are definitively uniform in order to truly characterize the effect of stimulating cartilage with CCEFs.

In the present study, we aimed to reproduce the experimental setup used by Brighton and coauthors but improve electric field calculations and verify field strength level and uniformity through finite element analysis (FEA) to definitively apply CCEFs with uniform field strength of 20 mV/cm to rabbit hyaline chondrocytes and bovine articular cartilage explants. We then examined the effect of the electrical stimulation on anabolism and catabolism in the chondrocytes and explants in the presence and absence of IL-1B. Cartilage anabolism was measured by quantifying the expression of the main components of cartilage matrix, Collagen-2 and Aggrecan, as well as tissue inhibitors of metalloproteinases (TIMPs), which are molecules that are naturally synthesized by chondrocytes to regulate the activity of MMPs (Martel-Pelletier et al. 2001). Catabolism was measured by quantifying the expression of MMPs, the inflammatory cytokine, Interleukin 6 (IL-6), and Aggrecanase-1 (ADAM-TS4), which is a metalloproteinase that cleaves aggrecan (Martel-Pelletier et al. 2001).

The goal of this study was to demonstrate that stimulating osteoarthritic cartilage with CCEFs that have verified uniformity leads to upregulation of anabolism and downregulation of catabolism in order to provide the foundation for a future clinical application of treating OA with CCEFs. This clinical application will be the development and usage of a device that can be directly applied to the knee joint of patients with OA using a brace-like configuration. The device will be designed so that it is controlled by wireless technology and can deliver uniform CCEFs with specified field strength to cartilage and the surrounding soft tissue. Future work will focus on engineering and optimizing the device’s ability to generate uniform CCEFs with different field strengths. Prototypes of this device are currently in development.

## Methods

### Experimental apparatus

**Fig. 1:**
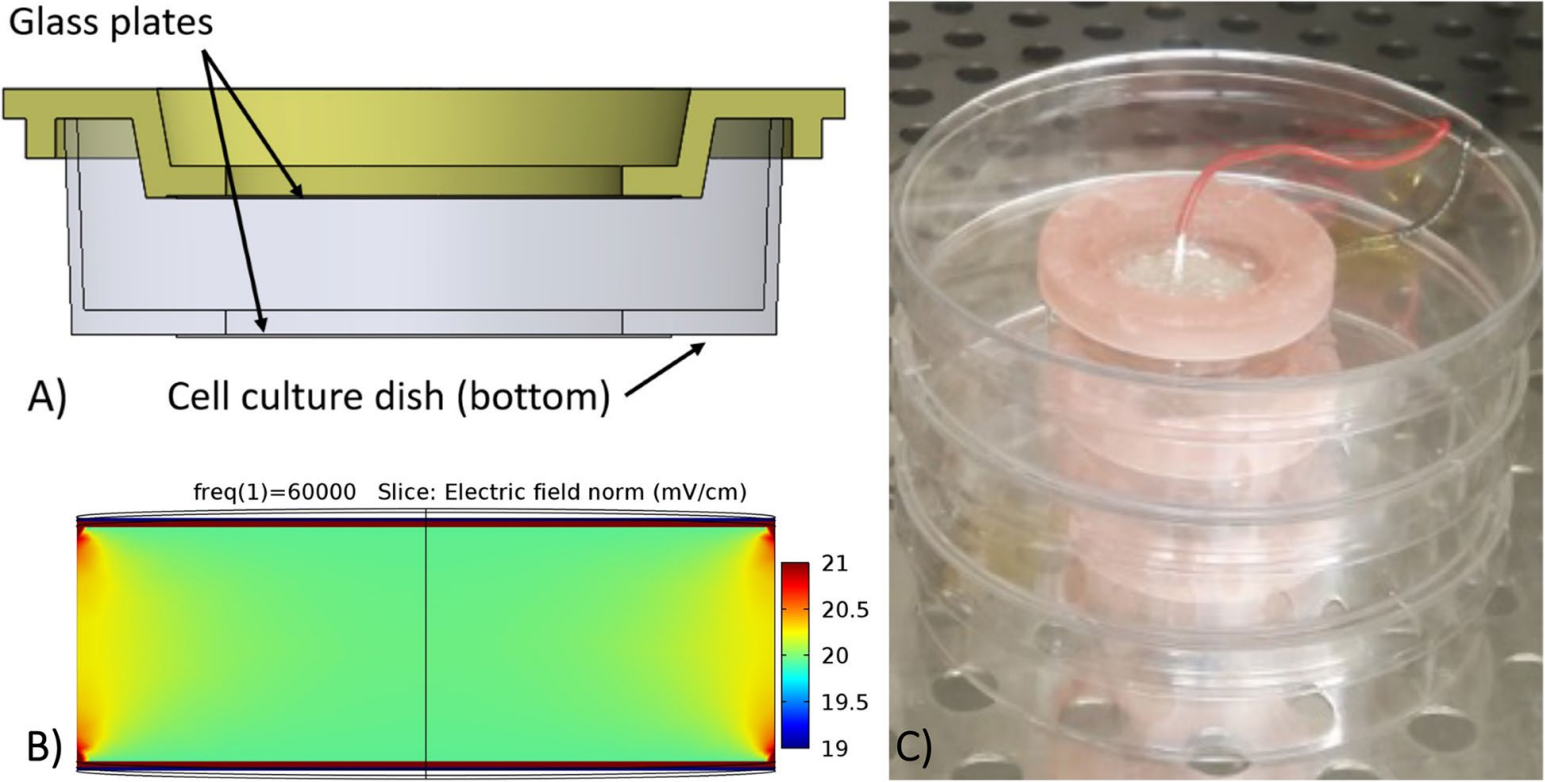
Experimental Apparatus. **A** A CAD rendering of the fabricated in-vitro exposure apparatus consisting of a cell culture dish and a custom lid with integrated glass coverslips to serve as the dielectric for capacitive stimulation. **B** Cross-section of the FEA model of the electric field generated within the in-vitro exposure apparatus. The field uniformity is within 5% of the desired value of 20 mV/cm in all regions within the dish. **C** A stack of manufactured culture dishes within an incubator showing the attached wires used to deliver the signal

Experimental apparatuses were engineered to model the capacitive fields as designed by Brighton and coauthors (Brighton et al. 2008; Wang et al. 2004; Brighton et al. 2013; Brighton et al. 2006). The apparatus consisted of a 60 mm cell culture dish with a glass coverslip (MatTek Corporation, P35-1184-S P35G-1.5–20-C.S) adhered to its bottom and a custom culture dish lid 3D-printed in biocompatible photopolymer resin (FormLabs Dental SG resin) with a matching glass coverslip adhered to it. Holes were made on the top and bottom of the culture dish in order for the glass coverslips to directly contact the culture medium and serve as the dielectric for capacitive stimulation. The glass coverslips were adhered to the culture dishes using biocompatible silicone (NuSil MED-4211) (Fig. 1A). Conductive epoxy (MG Chemicals 8331) was applied in a circular area with a diameter of 20 mm on the outer surface of both glass coverslips. Outputs of a power amplifier were connected to each layer of conductive epoxy, forming a parallel plate capacitor, in order to generate an electric field (Fig. 1C).

The input signal for generation of a sinusoidal electric field was provided by a waveform generator (Keysight 33500B). A MATLAB program was written to modulate the waveform generator in order to implement the desired duty cycle for CCEF exposure. The sinusoidal signal was amplified with a power amplifier (AE Techron 7224) in order to produce sufficient voltages required for the generation of a 20 mV/cm electric field. The amplitude of the applied signal corresponding to a uniform 20 mV/cm field strength was determined via FEA (COMSOL Multiphysics) based on the electrical properties of the conductive epoxy plates and culture medium and the geometry of the culture dish and coverslips and was verified by directly measuring the output of the power amplifier. This amplitude was 71.1 V and the frequency of the applied signal was 60 kHz. The FEA results demonstrate uniformity of the 20 mV/cm electric field (Fig. 1B).

#### Rabbit hyaline chondrocytes

Rabbit hyaline chondrocytes were enzymatically isolated from fresh cartilage and expanded in culture until a third passage in Dulbecco’s Modified Eagle Medium (DMEM) F12 + 10% fetal bovine serum (FBS). The chondrocytes were plated at a seeding density of 200,000 cells in 60 mm dishes within the experimental apparatuses. The chondrocytes were separated into four groups (*N* = 3/group): a) No CCEF (control) b) CCEF c) No CCEF + 10 ng/ml IL-1B and d) CCEF + 10 ng/ml IL-1B. Cells were treated for a total of 7 days with medium changed every 2 days. The duty cycle for the CCEF exposure was 1 h on followed by 4 h off for 24 h delivering active field effect for 4 h per day. Total RNA was isolated after 7 days of stimulation using RNeasy Mini Kit (Qiagen) and the following genes were tested using quantitative polymerase chain reaction (QPCR): MMP3, MMP9, TIMP1, TIMP2, ADAM-TS4, IL-6, and Glyceraldehyde 3-Phosphate Dehydrogenase (GAPDH) as the housekeeping gene. The primer pairs that were used are listed in Table 1. The delta-delta CT method was used to extract and analyze QPCR results. All gene expression values were normalized to the untreated group (control).

**Table 1:** QPCR Primer Sets for Rabbit Targets

GENE	FORWARD [L]	REVERSE [R]
GAPDH	CATCACTGCCACCCAGAAGA	GCCAGTGAGTTTCCCGTTCA
MMP3	GACTCCACTCACGTTCTCCAG	GCCAAAACATTTCCAGGTCCA
MMP9	CCTTTGAACACACACGACGTC	GGTACTCACACGCCAGAAGAA
ADAM-TS4	TCTTCAAGAACTTCCCAGGGC	ACATGGGGTGCCATCTATCAC
TIMP1	CCGGACAGACGCTAGAGAATC	GACAAGGTCGGAGTTGCAGAA
TIMP2	CCTTGGCTTTGTTCTGTGAGC	AGAGCGAGGCCATCTTTGAAA
IL-6	CGGCGGTGAATAATGAGACCT	TGCTGACCCTGGTGTTTTCTT

Primer pairs used in QPCR to quantify gene expression of rabbit hyaline chondrocytes. The forward and reverse sequences of each primer are listed

### Bovine articular cartilage explants

Freshly slaughtered bovine legs were procured from Green Village Packing Co. The legs were sterilized with betadine and placed in laminar air-flow hood for harvesting cartilage. The dissection was performed using sterile technique and full-thickness articular cartilage was excised and placed in a petri dish with saline. A 3 mm biopsy punch was used to create uniform bovine cartilage explants. These explants were washed 4 times for 10 min each with PBS (Phosphate-buffered saline) and 10% Antibiotic-antimicotic solution and then placed in DMEM F12 + 10% FBS. The explants were cultured for 2 days to ensure absence of contamination. The explants were then randomly segregated into groups (*N* = 9/group). Experimental grouping of explants and duty cycle for CCEF exposure was the same as the chondrocytes.

After 1 day of stimulation, tissue was homogenized using Mikro-dismembrator and total RNA was isolated using Trizol method. Gene expression of MMP3, Aggrecan, Collagen-2, and TIMP1 was tested with QPCR with GAPDH as the housekeeping gene. The primer pairs that were used are listed in Table 2. The delta-delta CT method was used to extract and analyze QPCR results. All gene expression values were normalized to the untreated group (control).

**Table 2:** QPCR Primer Sets for Bovine Targets

GENE	FORWARD [L]	REVERSE [R]
GAPDH	ACAGTCAAGGCAGAGAACGG	CCAGCATCACCCCACTTGAT
MMP3	AACCTTCCGATTCTGCTGTTG	GGTGTCTTCCTTGTCCCTTG
TIMP1	AGAGCGTCTGCGGATACTTC	TGTTCCAGGGAGCCACAAAA
AGGRECAN	CCCGACTGATGCTTCTATCCC	CGCACAGCTTCTGGTCTGTTG
COLLAGEN-2	TAAGGATGTGTGGAAGCCCGA	GGCTGAGGCAGTCTTTCATGT

Primer pairs used in QPCR to quantify gene expression of bovine articular cartilage explants. The forward and reverse sequences of each primer are listed

After one week of stimulation, bovine explants were fixed in zinc-buffered formalin for one week and embedded in paraffin for histology. Slides were stained with Safranin-O and Fast Green dye, commonly used to identify proteoglycan and Collagen-2, and to differentiate chondrocytes (red) from bone (green/blue).

### Statistical analysis

Statistical analysis of gene expression data was performed in Excel using one-way analysis of variance and Tukey–Kramer multiple comparison test with a significance level set at *p* <  = 0.05.

## Results

### Rabbit hyaline chondrocytes

**Fig. 2:**
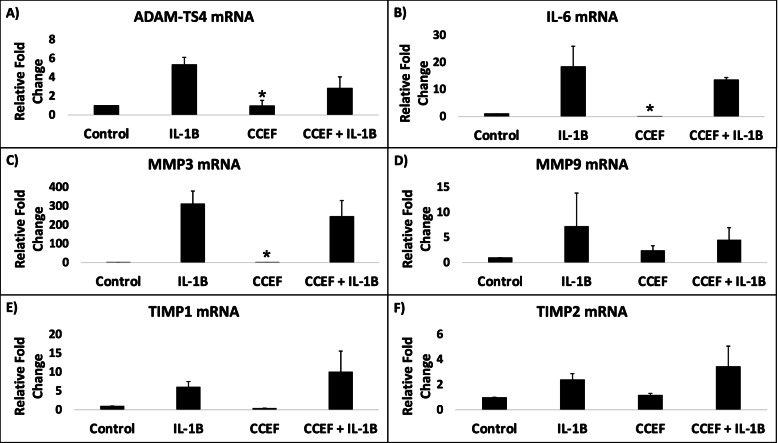
Gene Expression of Rabbit Hyaline Chondrocytes. Effect of CCEF stimulation of rabbit hyaline chondrocytes in the absence or presence of IL-1B for one week on gene expression of **A** ADAM-TS4, **B** IL-6, **C** MMP3, **D** MMP9, **E** TIMP1, and **F** TIMP2. A statistically significant difference (*p* <  = 0.05) from the IL-1B treatment group is denoted by an asterisk (*)

Treatment of rabbit hyaline chondrocytes with IL-1B for one week resulted in increased expression of ADAM-TS4, IL-6, MMP3, and MMP9 when compared to the untreated chondrocytes, however these increases were not found to be statistically significant. CCEF stimulation in the absence of IL-1B resulted in significantly decreased expression of ADAM-TS4, IL-6, and MMP3 compared to treatment with IL-1B alone. CCEF stimulation in the absence of IL-1B also resulted in decreased expression of MMP9 compared to treatment with IL-1B alone, but this decrease was not statistically significant. CCEF stimulation in the presence of IL-1B for one week resulted in decreased expression of ADAM-TS4, IL-6, MMP3, and MMP9 compared to treatment with IL-1B alone, however these decrements were not found to be statistically significant (Fig. 2A-D). CCEF stimulation in the presence of IL-1B led to upregulation of TIMP1 and TIMP2 compared to treatment with IL-1B alone, but these changes were not found to be statistically significant (Fig. 2E-F). Although the reported differences in gene expression between the IL-1B treatment group and combined CCEF and IL-1B treatment group were not statistically significant, they demonstrate a promising trend that should be further investigated.

### Bovine articular cartilage explants

**Fig. 3:**
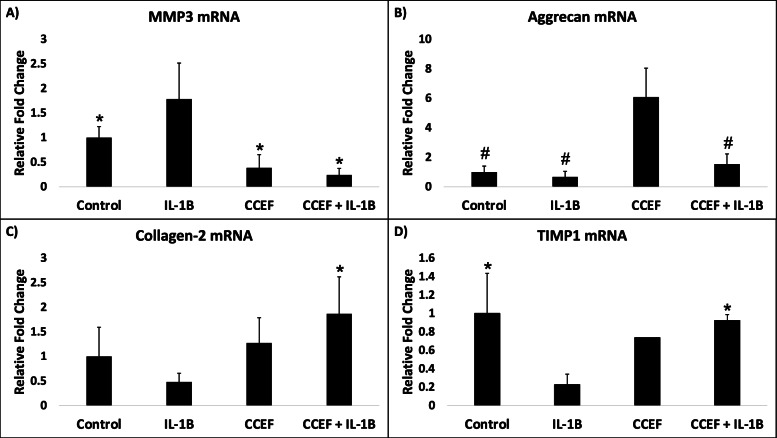
Gene Expression of Bovine Articular Cartilage Explants. Effect of CCEF stimulation of bovine articular cartilage explants in the absence or presence of IL-1B for one day on gene expression of **A** MMP3, **B** Aggrecan, **C** Collagen-2, and **D** TIMP1. A statistically significant difference from the IL-1B treatment group is denoted by an asterisk (*). A statistically significant difference from the CCEF treatment group is denoted by a (#)

Treatment of bovine articular cartilage explants with IL-1B resulted in significantly increased expression of MMP3 relative to the explants that received no treatment. CCEF stimulation of bovine articular cartilage explants in both the absence and presence of IL-1B resulted in significantly decreased expression of MMP3 compared to treatment with IL-1B alone. Stimulation in the absence and presence of IL-1B also resulted in an apparent decrease in MMP3 expression compared to the control group, however this decrease was not significant (Fig. 3A). Treatment of explants with IL-1B led to a decrease in expression of Aggrecan relative to the explants that received no treatment, however this decrease was not statistically significant. CCEF stimulation of explants in the absence of IL-1B resulted in a significant increase in expression of Aggrecan relative to no treatment, treatment with IL-1B, and simultaneous treatment with IL-1B and CCEF. CCEF stimulation in the presence of IL-1B resulted in an increase in Aggrecan expression relative to treatment with IL-1B alone, but this difference was not found to be statistically significant (Fig. 3B). Treatment of explants with IL-1B led to a decrease in Collagen-2 expression relative to the explants that received no treatment, however this decrease was not statistically significant. CCEF stimulation in the presence of IL-1B led to a significant upregulation of Collagen-2 expression relative to treatment with IL-1B alone (Fig. 3C). Treatment of explants with IL-1B led to a significant decrease in TIMP1 expression when compared to the explants that received no treatment. CCEF stimulation in the presence of IL-1B resulted in a significant increase in TIMP1 expression relative to treatment with IL-1B alone (Fig. 3D).

**Fig. 4:**
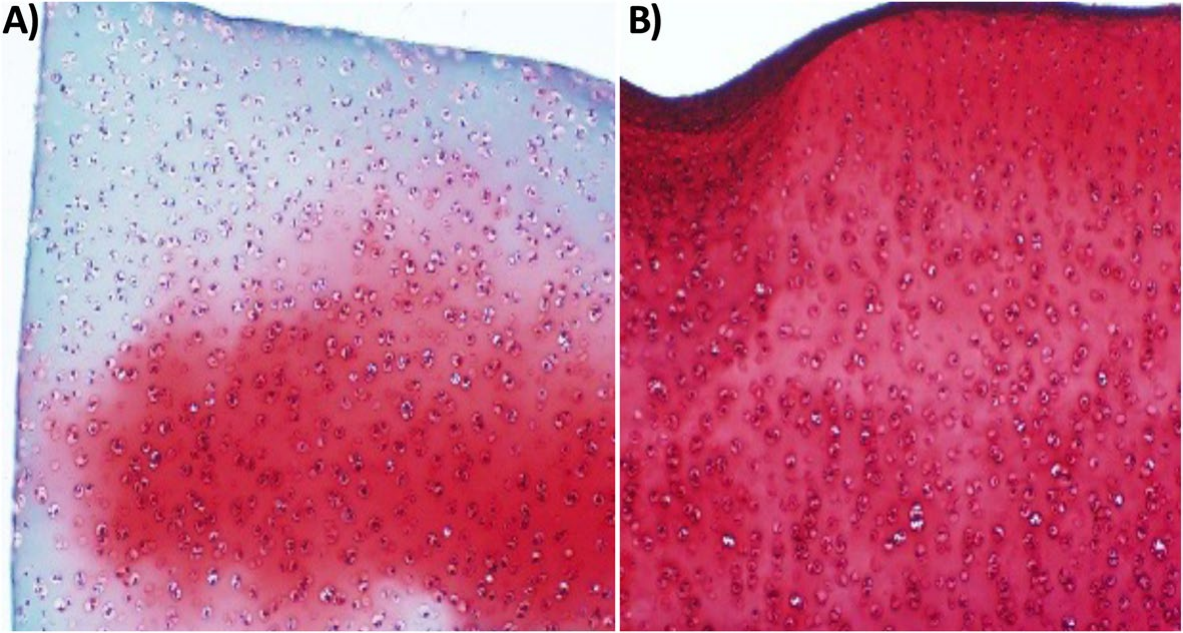
Histological Analysis of Aggrecan in Bovine Articular Cartilage Explants. Histological analysis of aggrecan in bovine articular cartilage explants **A** treated with IL-1B alone and **B** treated with CCEF stimulation and IL-1B for one day

Histological analysis of aggrecan demonstrated depletion of aggrecan in bovine cartilage explants treated with IL-1B for one day (Fig. 4A). Bovine articular cartilage explants that underwent CCEF stimulation and treatment with IL-1B for one day demonstrated retention of aggrecan throughout the entire thickness of the tissue (Fig. 4B).

## Discussion

The results of this study demonstrate the potential of CCEF stimulation to reverse the degenerative changes of OA and promote matrix deposition in articular cartilage. Bovine articular cartilage explants simultaneously treated with IL-1B and CCEFs had significantly more gene expression of matrix proteins and TIMP1 and significantly less gene expression of MMP3 than explants treated with IL-1B alone. Although the application of IL-1B and CCEFs to rabbit hyaline chondrocytes did not yield statistically significant differences in gene expression, there is a clear trend showing that treatment with IL-1B resulted in elevated expression of inflammatory markers and decreased expression of matrix components while simultaneous treatment with IL-1B and CCEFs resulted in a relative decrease in expression of inflammatory markers and increase in expression of matrix components. These results are consistent with the results of the studies performed by Brighton and coauthors (Brighton et al. 2008; Wang et al. 2004; Brighton et al. 2013; Brighton et al. 2006). The present study shows that CCEFs with verified uniformity and field strength of 20 mV/cm are sufficient to produce a similar increase in cartilage matrix anabolism and decrease in cartilage matrix catabolism in the presence of IL-1B (Brighton et al. 2008; Wang et al. 2004; Brighton et al. 2013; Brighton et al. 2006).

FEA is a numerical method based on established physics that is used to accurately estimate the parameters of CCEFs generated by experimental setups. Although it would be ideal to directly measure the generated electrical fields, it is difficult to do this accurately. It is especially difficult to measure electric fields applied to cells or tissue within culture medium like in the present study because the probe would have to enter the culture medium. Studies that apply electric fields to cells *in-vitro* rarely include measurement of the electric fields due to this difficulty. FEA is a sufficient alternative to direct measurement for determining electric field parameters (Meneses et al. 2022). Brighton and coauthors did not describe use of FEA or other analytical methods in any of their studies to demonstrate that the CCEFs generated by their setup actually achieved the reported parameters (Brighton et al. 2008; Wang et al. 2004; Brighton et al. 2013; Brighton et al. 2006). The innovation of the present study was improving the experimental setup previously used by Brighton and coauthors by recreating the setup and performing FEA to demonstrate that the generated electric fields were uniform and had the desired field strength. Future studies that use the experimental setup described in this study to apply CCEFs to cartilage should also use FEA or develop a method of accurate electric field measurement to ensure that the generated electric fields are uniform and have the desired strength.

We ultimately chose to use two different *in-vitro* models of OA to investigate the effect of CCEF stimulation on metabolism of osteoarthritic cartilage. Rabbit hyaline chondrocytes in monolayer culture treated with IL-1B were initially chosen in order to analyze and understand the effect of CCEF stimulation on chondrocytes that are isolated from tissue. We decided to expand the study to include a model of OA that more closely simulates cartilage *in-vivo* once we observed that application of uniform CCEFs to isolated chondrocytes treated with IL-1B resulted in increased anabolism and decreased catabolism relative to isolated chondrocytes treated only with IL-1B. This model consisted of full-thickness bovine articular cartilage explants treated with IL-1B. We believed that it was important to include this model to determine whether application of uniform CCEFs also results in increased anabolism and decreased catabolism in whole cartilage and not strictly in isolated chondrocytes.

The main limitation of this study was that the modified culture dishes within the experimental apparatuses were not suitable for culturing cell or tissue for long periods due to a lack of a well-sealed system and resulting increased probability of contamination. The modified culture dishes were not made of a material capable of serving as a dielectric for applying an electric field to the culture medium and cells or tissue within the dish. Glass is capable of serving as a dielectric for this application, so glass coverslips were adhered to the top and bottom of the culture dishes and holes were made in the dishes in order for the coverslips to directly contact the culture medium (Brighton et al. 2008). We believe that a complete seal was not achieved even though the glass coverslips were tightly adhered to the culture dishes using biocompatible silicon. This lack of a complete seal increased the chances of contamination. We only used the culture dishes that appeared free of contamination for analysis. Each different culture dish containing rabbit hyaline chondrocytes that received the same treatment served as one biological replicate. Each different culture dish with bovine articular cartilage explants contained three different explants or three biological replicates. There were three or less remaining biological replicates of rabbit hyaline chondrocytes per treatment group after selecting only the culture dishes that were not contaminated while there was still a sufficient number of biological replicates of bovine articular cartilage explants since there were three explants per culture dish. The low number of biological replicates of rabbit hyaline chondrocytes may have contributed to the lack of statistically significant differences in gene expression. Despite the low number of biological replicates, we observed a trend in gene expression showing increased anabolism and decreased catabolism in the chondrocytes treated with both CCEF and IL-1B relative to those treated with just IL-1B. We hypothesize that this trend would be more remarkable and differences in gene expression would be statistically significant if more biological replicates of the rabbit hyaline chondrocytes were analyzed. Future work should improve the design of the experimental apparatus to include a complete well-sealed system for culturing cell or tissue that is capable of applying electric fields. This improvement would greatly reduce the probability of contamination and allow for analysis of a higher number of biological replicates.

Another possible explanation for the lack of statistically significant differences in the gene expression of matrix proteins, TIMPs, MMPs, IL-6, and ADAM-TS4 between rabbit hyaline chondrocytes treated with IL-1B and those treated with IL-1B and CCEFs is the dedifferentiation of chondrocytes in monolayer culture. Dedifferentiation is the process in which cultured primary chondrocytes lose their phenotype and ability to secrete cartilage matrix (Ma et al. 2013). This loss of phenotype may disrupt the natural response of chondrocytes to electrical stimulation and may partially explain why CCEF stimulation of chondrocytes did not produce the same significant increase in anabolism and decrease in catabolism as stimulation of the explants.

We cultured the rabbit hyaline chondrocytes for seven days and observed the contamination before beginning culture of the bovine articular cartilage explants. We decided to culture the bovine articular cartilage explants for a shorter duration of one day to reduce the probability of contamination. Improving the design of the experimental apparatus to include a complete well-sealed system would allow for longer duration of culture and treatment with CCEFs. Brighton and coauthors applied IL-1B and CCEF stimulation to bovine articular cartilage explants for one week and showed highly significant increases in Aggrecan and Collagen-2 expression in the explants stimulated by CCEFs both in the absence and presence of IL-1B (Brighton et al. 2006). In another study, Brighton and coauthors applied IL-1B and CCEF stimulation to human articular cartilage explants for one week and showed highly significant increases in Aggrecan and Collagen-2 mRNA expression as well as decreases in MMP1, MMP3, MMP13, and ADAM-TS4 mRNA expression in explants that were treated with CCEFs and IL-1B relative to explants that were just treated with IL-1B (Brighton et al. 2008). Based on the results of these studies, it is possible that stimulation of both bovine articular cartilage explants and rabbit hyaline chondrocytes for longer durations than the duration of stimulation in the present study would produce more significant results. However, the relatively increased gene expression of factors associated with cartilage anabolism and decreased expression of factors associated with cartilage catabolism in bovine articular cartilage explants after only one day of CCEF stimulation highlights the potential of this technique as an effective treatment for OA.

The present study analyzed gene expression and Aggrecan histology at only one time point. It would be interesting to characterize the time course of the metabolic response of cartilage to CCEF stimulation by analyzing gene expression and Aggrecan histology at several different time points. Future studies should improve the experimental apparatus to reduce contamination and apply uniform CCEFs with a verified field strength of 20 mV/cm to rabbit hyaline chondrocytes and bovine articular cartilage explants for shorter and longer durations than seven days. These prospective studies have the potential to examine the time course of the effect of CCEFs on cartilage metabolism and determine the optimal duration of CCEF stimulation.

A field strength of 20 mV/cm may not be the optimal field strength for CCEF stimulation of articular cartilage. Brighton and coauthors performed experiments to determine the optimal field strength for CCEF stimulation of fetal bovine chondrocytes and determined that it was 20 mV/cm (Wang et al. 2004). However, the calculations performed by Brighton and coauthors to determine field strength were unclear and field strength was not confirmed through analysis, so their conclusion regarding optimal field strength should be verified in future studies. These studies should produce CCEFs with several different field strengths, perform FEA to ensure accuracy and uniformity of the field strengths as was done in the present study, and compare the effects of the different field strengths on cartilage metabolism to determine whether a field strength of 20 mV/cm is truly optimal. Brighton and coauthors also experimentally determined that the best CCEF stimulation protocol consisted of 30 min of continuous stimulation (100% duty cycle) followed by pulsed (1 h on, 5 h off) 50% duty cycle (1 min on, 1 min off) each day (Brighton et al. 2008; Wang et al. 2004). It would be beneficial for future studies to verify this conclusion or find the optimal stimulation protocol by stimulating cartilage with CCEFs that have verified field strengths and uniformity in addition to varied timing protocols and duty cycles.

## Conclusion

The present study improved upon the CCEF stimulation of articular cartilage initially described by Brighton and coauthors (Brighton et al. 2008; Wang et al. 2004; Brighton et al. 2013; Brighton et al. 2006) by performing FEA to verify that the CCEFs are uniform and have a field strength of 20 mV/cm. The application of these CCEFs to rabbit hyaline chondrocytes and bovine articular cartilage explants resulted in upregulation of matrix anabolic factors and downregulation of matrix catabolic factors in the absence and presence of an inflammatory cytokine, IL-1B, that was used to model OA. These results are consistent with the outcomes of the studies by Brighton and coauthors (Brighton et al. 2008; Wang et al. 2004; Brighton et al. 2013; Brighton et al. 2006), providing greater evidence for the therapeutic potential of CCEF stimulation of osteoarthritic cartilage. Demonstrating that CCEFs with defined field strengths and proven uniformity may reverse the progression of OA brings CCEF stimulation of articular cartilage closer to use in clinical studies.

## Data Availability

The datasets used and analyzed during the current study are available from the corresponding author on reasonable request. The corresponding author can be reached by email at knoruzi@northwell.edu or knoruzi@student.nymc.edu.

## Abbreviations

OA: Osteoarthritis
CCEF: Capacitively Coupled Electric Field
IL-1B: Interleukin1-B
MMP: Matrix Metalloproteinase
FEA: Finite Element Analysis
TIMP: Tissue Inhibitor of Metalloproteinase
IL-6: Interleukin 6
ADAM-TS4: Aggrecanase 1
DMEM: Dulbecco’s Modified Eagle Medium
FBS: Fetal Bovine Serum
QPCR: Quantitative Polymerase Chain Reaction
GAPDH: Glyceraldehyde 3-Phosphate Dehydrogenase
PBS: Phosphate-Buffered Saline
